# Intentional Forgetting in Organizations: The Importance of Eliminating Retrieval Cues for Implementing New Routines

**DOI:** 10.3389/fpsyg.2018.00051

**Published:** 2018-02-01

**Authors:** Annette Kluge, Norbert Gronau

**Affiliations:** ^1^Industrial, Organisational and Business Psychology, Faculty of Psychology, Ruhr University Bochum, Bochum, Germany; ^2^Business Informatics, Processes and Systems, University of Potsdam, Potsdam, Germany

**Keywords:** change management, multi-actor routines, business processes, knowledge management, organizational memory, situational strength

## Abstract

To cope with the already large, and ever increasing, amount of information stored in organizational memory, “forgetting,” as an important human memory process, might be transferred to the organizational context. Especially in intentionally planned change processes (e.g., change management), forgetting is an important precondition to impede the recall of obsolete routines and adapt to new strategic objectives accompanied by new organizational routines. We first comprehensively review the literature on the need for organizational forgetting and particularly on accidental vs. intentional forgetting. We discuss the current state of the art of theory and empirical evidence on forgetting from cognitive psychology in order to infer mechanisms applicable to the organizational context. In this respect, we emphasize retrieval theories and the relevance of retrieval cues important for forgetting. Subsequently, we transfer the empirical evidence that the elimination of retrieval cues leads to faster forgetting to the forgetting of organizational routines, as routines are part of organizational memory. We then propose a classification of cues (context, sensory, business process-related cues) that are relevant in the forgetting of routines, and discuss a meta-cue called the “situational strength” cue, which is relevant if cues of an old and a new routine are present simultaneously. Based on the classification as business process-related cues (information, team, task, object cues), we propose mechanisms to accelerate forgetting by eliminating specific cues based on the empirical and theoretical state of the art. We conclude that in intentional organizational change processes, the elimination of cues to accelerate forgetting should be used in change management practices.

## Organizational learning and the supplemental need for forgetting

For a long time, a dominant strategic view has argued for organization's knowledge and learning capabilities as a main source of competitive advantage (Hamel and Prahalad, 1993; Spender, 1996). Learning has been valued because it assists a firm in outdoing its competitors (Pedler et al., 1989; Frey, 1990; Dodgson, 1991; Parke, 1991; Hamel and Prahalad, 1993). Learning organizations as instruments enable continuous development via a system of procedures and routines, i.e., corporate structures that facilitate collective learning. Thus, learning requires changing organizational objectives, competencies, structures, and routines to adapt to a changing environment (Kluge and Schilling, 2003).

Since these early days of the postulation of the learning organizations, organizations indeed have learned and experienced a lot. Lessons-learned, experience and knowledge in general has been acquired, disseminated and stored with the support of knowledge management systems of different forms, e.g., IT-based or socially shared (Schilling and Kluge, 2009). That means that in modern-day organizations, huge amount of knowledge haven been gathered, processed and stored in organizational memory and are continuously enlarged. Especially technical limits in terms of limited storage have not been an issue for several decades (Lasica, 1998; Brynjolfsson and McAfee, 2014). The costs and efforts involved in storing and copying information are low (Whelan and Teigland, 2013). Organizational processes such as “Exploration and Exploitation” (March, 1991), which describes the search, acquisition and elaboration of new information, the intensive and excessive use of information, and the evaluation of information as an important resource, have led to a continuous increase in available and recallable stored knowledge (Blaschke and Schoeneborn, 2006; Miller and Martignoni, 2016).

As knowledge acquisition and sharing as well as learning have been acknowledged and valued as important processes on individual, team and organizational level (Huber, 1991; Crossan et al., 1999; Schilling and Kluge, 2009; Putz et al., 2013), we claim that now is it time to address the next level of successful learning, which is forgetting from our perspective. We thereby extend the view of an evolving organization that learns from experience by the notion of, e.g., Argyris and Schön (1978), Crossan et al. (1999), Fiol and Lyles (1985), Kluge and Schilling (2003). We propose that forgetting is an important process, as a high amount of available and stored knowledge can also lead to difficulties in interpreting information and might impede the evaluation of alternative ways to reach strategic goals (Lipshitz and Strauss, 1997). This will also result in uncertainty about which goals can be achieved under consideration of all knowledge (Grote, 2009) or to problems with establishing connections and causalities (Kareev, 2000) and patterns among the noise of all of the available knowledge.

We extent the view by adding the demand for not applying all experiences at the same time, by implementing processes named forgetting in general and on possibilities and means to facilitate adaptation to current situational and environmental demands by the means of intentional forgetting in particular.

The objective of the paper is to elaborate on the use of individual forgetting as valuable concepts for organizations as well. We argue from a basic human memory and cognitive psychology perspective and transfer the concept of intentional forgetting to the organizational context of learning and change.

In this paper, we propose that in order to cope with the large amount of knowledge stored in organizations, “forgetting,” as an important, successfully evolved human characteristic (Wixted, 2004, 2005; Klein et al., 2010), can also be transferred and used by an organization as a socio-digital system. Forgetting is not a malfunction in human information processing (Wixted, 2004, 2005; Roediger et al., 2010), but is rather an essential adaptive function to overwrite, suppress and sort out information that is no longer up to date (Bjork, 1998). The human memory does not delete obsolete knowledge, but is able to not recall it and to suppress it. *If the environment changes, adaptability is required, meaning that previous objectives need to be forgotten in order to focus on currently relevant objectives* (Altmann and Gray, 2002; Roediger et al., 2010). This assumption is also relevant for organizations in changing environments. Organizations change their goals and strategies when the market, customers, technologies, regulations etc. change, and subsequently need to forget previous objectives and solutions in order to focus on currently relevant objectives.

In this respect, forgetting impedes the recall of obsolete knowledge in individuals (Schooler and Hertwig, 2005) and is proposed to be a useful concept in organizations as well. The adaptive function of human information serves purposes of future decision making and future evaluations (Klein et al., 2010). The term “Intentional Forgetting” describes the process which humans use to control and regulate their memories (Bjork et al., 1998; Payne and Corrigan, 2007; Lehman and Malmberg, 2011). Nevertheless, although organizations do actively use the advantage of learning, they do not actively use and implement the human *advantage* of forgetting as a competitive advantage, as current organizational theories are unable to supports its implementation (Suddaby et al., 2011).

The paper has four parts:

First, we summarize the research on organizational forgetting from an organizational and management science perspective and introduce the distinction between accidental and intentional forgetting.
Second, we present the state of the art on forgetting theories on an individual and organizational level, starting with basic cognitive psychology research, proceeding with industrial and engineering psychology and closing with forgetting in research on business processes and knowledge management. Based on basic research, we stress that forgetting depends on the absence of retrieval cues and that the absence of retrieval cues is central to impeding the recall of memory items.
Third, be propose that intentional forgetting is relevant in the organizational context of implementing routines that differ from the routines that have been performed and executed with high levels of proceduralizations.
Forth and finally, based on the theory of forgetting caused by missing retrieval cues, we introduce a system for cue classification and develop propositions, how to support forgetting in an organizational context by intentionally eliminating retrieval cues of different kinds.

## Organizational forgetting and its facets

The review regarding intentional forgetting in organizations was conducted based on guidelines of Tranfield et al. (2003) on how to undertake a systematic review by searching leading electronic data bases including peer-reviewed publications, conference proceedings and Internet Sources listed in GoogleScholar, PsycArticles, PsyINFO. Psyndex (via EBSCO) using the following keywords: organis(z)ational forgetting, intentional forgetting in organis(z)ations, organis(z)ational unlearning, organis(z)ational ignorance, knowledge management and forgetting, managing forgetting. Publications that were found using the initial key word “organization and forgetting” and “intentional forgetting in organis(z)ations” also provided synonyms which were then used as key words. All together 246 publications were found. The 40 publications cited below were included via an examination of the abstracts and in-depth reviews in order to identify core contributions. Publications including case studies that only applied core concepts in a particular setting were not included as they were not contributing to a theoretical differentiation between concepts of organis(z)ational intentional forgetting and unlearning. Finally, the included core publications were clustered according the concepts of unlearning, accidental and intentional forgetting, selective forgetting, ignorance and conscious not-knowing, rearrangement and deleting.

Our findings suggest that some rather isolated conceptual thoughts concerning forgetting have been already developed, and address forgetting in different ways (Sinkula, 2002; Easterby-Smith and Lyles, 2003, 2011; Martin de Holan and Phillips, 2004; Becker, 2005; Akgün et al., 2006; Casey and Olivera, 2011; Martin de Holan, 2011). Nevertheless, these mainly theoretical concepts are not linked to one another, and have not been empirically assessed. These concepts are not linked in a sense that there was no attempt to build an integrative model or theory that allows deriving hypotheses and propositions about how to use or avoid forgetting so far. Instead the field has become more diverse and by the coexistence of different connotations of forgetting (e.g., as accidental, unintended or intentional).

So far, research on organizational forgetting processes has addressed the ideas of unlearning, replacing, ignoring, rearranging or deleting.

*Unlearning* (Hedberg, 1981; Huber, 1991; Tsang and Zahra, 2008; Fiol and O'Connor, 2017a; Reese, 2017; Starbuck, 2017; Tsang, 2017b; Visser, 2017), in the sense of discarding and replacing old routines (Huber, 1991), is assumed to support the objective to install new routines (Tsang and Zahra, 2008). In this context, forgetting refers to the unlearning of routines which no longer serve the organizational objectives and to the “installation” of new routines which do support the organizational goals.

*Selective forgetting, ignorance and the consciousness of Not-Knowing* (Roberts, 2013, *ignorance about existing knowledge*) means that individuals and teams in organizations have actively chosen to no longer invest resources in the storage of a defined set of information. In this context, forgetting means the suppression of temporarily irrelevant information.

*Voluntary Forgetting* (Martin de Holan, 2011) refers to the facilitation of change, especially when current knowledge is perceived as an obstruction and a competitor to new knowledge.

*The rearrangement of information* (Martin de Holan, 2011) has the purpose of abstracting and generalizing existing information and relieving it from details. If an organization wants to rearrange, it needs to decide on possible futures and contexts, as future contexts have to be anticipated.

*Deleting* (Akgün et al., 2006) as a process of organizational forgetting means that an organization radically cuts off obsolete, useless or even false/untrue information. This can also mean cutting off information that has become irrelevant, because the environment has changed or the information was later proven to be false.

**Figure 1:**
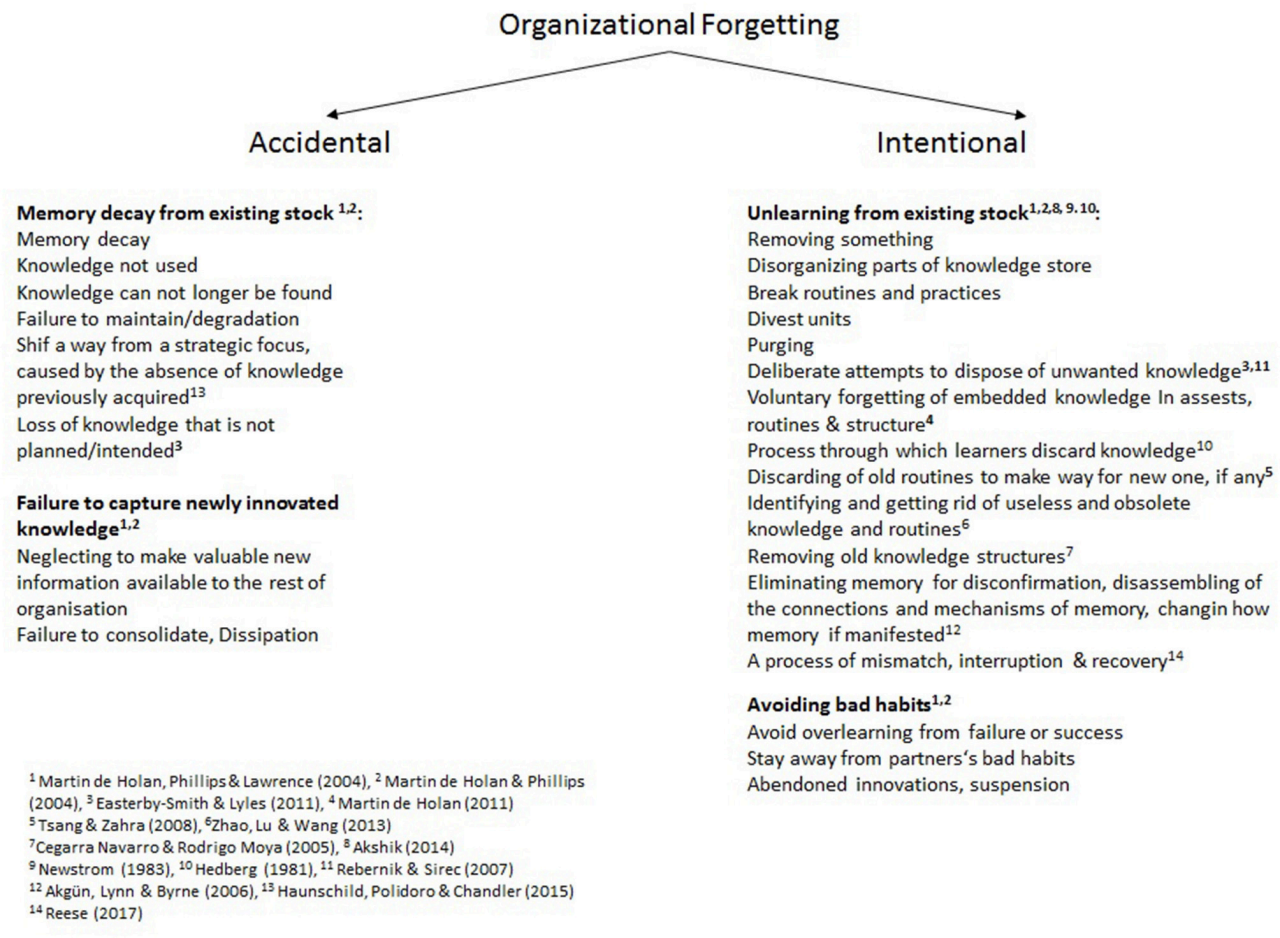
Overview of central concepts of accidental and intentional forgetting.

Taking these and additional concepts together, organizational forgetting can be accidental or intentional (Figure 1).

Why not referring to “Unlearning”?

In particular, the *unlearning* concept has recently been discussed and reviewed. A current review of the unlearning concept and its integration into the organizational learning models can be found in Visser (2017). The current debate addresses the following topics:

Questions have been raised regarding:

- whether the concept of unlearning can be subsumed under the concept of learning; whether it is a precondition of learning, or
- whether learning and unlearning are distinct types of organizational change (Nguyen, 2017; Rupčić, 2017);
- whether unlearning is one type of forgetting (see Figure 1 and Nguyen, 2017); and
- whether unlearning is the discarding of obsolete and misleading knowledge or the abandoning of any knowledge without evaluating the status of the knowledge (Hislop et al., 2014; Nguyen, 2017).
- Unlearning is mainly considered as an organizational reaction to a threat or serious crisis (Starbuck, 2017), turbulence (Morais-Storz and Nguyen, 2017) or exogenous disruption or destabilizing triggers (Fiol and O'Connor, 2017a,b).
- One of the main themes in the unlearning debate is the alternation between unlearning and learning, in which an external adverse stimulus triggers unlearning.

However, the concept of unlearning was also criticized in the management science community. A harsh critique on the use of the psychological concept of unlearning can be found in Howells and Scholderer (2016), commented on by Tsang (2017a). Howells and Scholderer (2016) argue that the paper by Hedberg (1981)—as the origin of the line of the work regarding unlearning—“used experimental psychology articles as authorities in support of the assertion that unlearning is an empirically warranted process” (p. 448). The experiments cited by Hedberg (1981) address extinction and not unlearning (Howells and Scholderer, 2016). There is no logical link between the process described in the literature cited by Hedberg in pair-associated learning and a phenomenon a lot more complex as learning and forgetting in organizations. It is assumed that the authority of a review of the psychological literature is incorrectly attached to Hedberg's article (by Nystrom and Starbuck, 1984). Howells and Scholderer (2016) further argue that the reversal of learning by the process of unlearning is not supported by the cited psychology articles but was asserted to be possible and manageable by other authors such as Nystrom and Starbuck (1984). Howells and Scholderer (2016) summarize that there is no evidence of an independent precedent process to learning that deletes knowledge and aids the acquisition of new knowledge. According to Howells and Scholderer (2016), the model of unlearning has no explanatory value and is unnecessary, because alternatives and unproblematic concepts are available. In that respect, we refrain from using “unlearning” and present the commonly accepted alternatives from basic psychological research in the section on the state of the art of individual and organizational forgetting.

Therefore, in contrast to the debate on unlearning, *our approach to forgetting in organizations proposes processes that deliberately impede the recall of certain organizational memory items, and do not provide these memory items and information elements in the case of a certain query in order to support an organization's changed strategic goal achievement*. Our understanding of forgetting overlaps to some extent with Martin de Holan and Phillips' (2004) term “managed unlearning,” which was defined in the sense that “managers worked to forget established knowledge that was, or was perceived to be, a barrier to increased organizational effectiveness” (Martin de Holan and Phillips, 2004, p. 1611). Our definition seems to overlap with Grisold et al. (2017) thoughts on reducing the influence of old knowledge on cognitive and behavioral processes, the idea of Hislop et al. (2014) to stop using knowledge. Our definition differs as we do not assign the terms “established” “old,” and “new” to the concept of knowledge. Our definition is not about “old,” or even “obsolete” vs. “new,” but about relevance at a certain point in time. Memory research from cognitive psychology does not assign a value (as “obsolete”) to memory items. Memory items are distinguished according to their storage and retrieval strength as will be outlined below.

Additionally, our understanding differs from that of Martin de Holan and Phillips (2004) in two relevant aspects:

- First, we address the *required activities* to eliminate retrieval cues in order to achieve successful forgetting, and
- Second, we do not address new or innovative knowledge, but rather *concentrate on the suppression of existing organizational memory and the retrieval processes*, which are suppressed.

In this respect, we see forgetting not as a means to “clear a space” or “make way” for new knowledge, but as a means to impede the recall of knowledge that is theoretically available, but is not supposed to be used as it hinders the achievement of new objectives. This knowledge does not need to be deleted, overwritten and sorted out (as compared to a computer system), but merely has to be prevented from being recalled.

### The analogy between human and organizational memory and the aspect of adaptation

Our elaborations build on the analogy of the human memory and forgetting processes on the one hand and on contingency theory as well as fit-theories of organizations (Donaldson, 1993; Huber, 2011) on the other. It is assumed that organizational effectiveness depends on the fit between the internal organizational attributes, e.g., routines, and the conditions (threats as well as opportunities) of the external environment (Huber, 2011). If internal organizational and external environmental variables change, organizations need to adapt with respect to strategy, structure, people, technology and processes (Jones and Bouncken, 2008; Huber, 2011).

The objective of our approach is to support the transition to a new, more appropriate organization-environment fit (Miles et al., 1974; Venkatraman and Camillus, 1984; Volberda et al., 2012) triggered by environmental changes. In our approach wetransfer the beneficial mechanisms of human forgetting processes to the organizational context of implementing an adapted business process (a multi-actor routine), especially knowledge intensive business processes. Our perspective uses the human memory as an example, model and “best practice” on how to deal with an unlimited amount of stored knowledge, instead of perceiving humans as “faulty” compared to technical devices. In this respect, our approach to organizational forgetting as a process of adaptation to new objectives in a changed environment draws on the concepts of *human* and *non-human* storage bins (Huber, 1991; Walsh and Ungson, 1991; Cross and Baird, 2000) such as actors (individual human memory/storage bin) and the individuals performing routines (organizational memory and non-human storage bin).

## State of the art of forgetting theories on the individual and organizational level

In *cognitive psychology research*, forgetting describes the observation that we can no longer recall something that we used to be able to recall (Tulving, 1974; Cubelli, 2010; Wixted, 2010). Forgetting is the opposite of recall (Cubelli, 2010). It is a process of adaptation (Nairne and Pandeirada, 2008) and is therefore viewed by psychological science as a precondition for successful learning and recall (MacLeod, 1998). Although human memory is characterized by unlimited storage capacity of memory items stored in long-term memory (Bjork and Bjork, 1992; Kirschner, 2002; Storm, 2011), adaptability is necessary, as the past never repeats itself, at least not in exactly the same way. Therefore, it would not be of great value to humans to store exact copies of earlier experiences. Memories are valuable because the past supports humans in the present to make plans for the future (Nairne and Pandeirada, 2008; Klein et al., 2010).

In order to decide between different behavioral options, humans do not need to remember all details of an experience. Given the assumption that memory items serve the purpose of dealing with the present and anticipating the future, the advantage of forgetting becomes visible: a less detailed and less perfect memory of a past experience improves our capability to draw conclusions (Schooler and Hertwig, 2005) and to detect causal relationships (Kareev, 2000; Nairne and Pandeirada, 2008). In this respect, human memory proves to be sensitive to the probability that a past incident will be relevant in the future. The human brain “bets” that if the frequency with which a memory item is recalled from memory decreases, the likelihood that the memory item will be recalled in the future will also decrease (Schooler and Hertwig, 2005). As the information processing of unneeded memory items is costly, it is more favorable for the memory system to forget seldom used memory items (Schooler and Hertwig, 2005).

Theories that explain forgetting can be distinguished into theories that focus on decay, interference, retrieval, cue overload, cue availability, as well as on consolidation and repression (Nairne and Pandeirada, 2008; Roediger et al., 2010) and into theories addressing intentional forgetting (Johnson, 1994).

Theories of decay refer to forgetting as a spontaneous, autonomous process that emerges over time and depends on time (Nairne and Pandeirada, 2008; Roediger et al., 2010). Interference theory assumes that forgetting occurs because other incidents interfere with the encoding of memory items (Roediger et al., 2010) and consolidation of these memory items is impaired (Nairne and Pandeirada, 2008). Consolidation is the progressive process of stabilization of a memory trace after the acquisition of the memory item (Dudai, 2002).

Interference can occur in two ways: first, as a detrimental effect that imposes new learning items on already acquired items and their memory trace, and second, as an effect with an impact on the retrieval cues (Nairne and Pandeirada, 2008). As such, it is proposed that recall is triggered by retrieval cues and is *cue-driven*. Forgetting results because:

- the association between retrieval cues and memory item is unlearned and subsequent activity leads to weakening of the cue-target association (Nairne and Pandeirada, 2008, p. 186), or
- a retrieval cue is linked to many different memory items through additional learning (cue overload) and therefore every additionally learned cue association weakens the existing cue association (Nairne and Pandeirada, 2008; Roediger et al., 2010). A cue is overloaded when it has less diagnostic value for a particular memory item, because more memory items are summed up under that cue.

In addition to interference, forgetting results from changing cue conditions. If cues that are needed for recall are not present in a situation, cue-dependent forgetting follows (Tulving, 1974). Cue-dependent forgetting is neither a consequence of decay or a fading memory trace nor a consequence of a weakened link between cue and memory item. Rather, humans forget because an appropriate retrieval cue is missing (Nairne and Pandeirada, 2008). Besides the more passive form of forgetting, there are also active mechanisms to inhibit and suppress recall. Due to active suppression, a memory trace is temporarily not reachable (Nairne and Pandeirada, 2008). This process is called *retrieval-induced forgetting* (Nairne and Pandeirada, 2008; Harris et al., 2010; Roediger et al., 2010). The capability to actively suppress memory items is essential to avoid cognitive overload and to show appropriate reactions (Roediger et al., 2010).

In their review papers, Nairne and Pandeirada (2008), Roediger et al. (2010) as well as Anderson and Hanslmayr (2014) elaborate on *motivated forgetting*, a mechanism to block fear-inducing memories from becoming conscious, as well as on *intentional* and *directed* forgetting. These three concepts have in common that they assume that individuals have executive control processes directed at minimizing the accessibility of memory items and stopping strong habitual responses to cues (Anderson and Green, 2001; Aguirre et al., 2017; Hu et al., 2017). In a current paper by Hu et al. (2017) for example, the authors claim that prior research has shown that suppressing the retrieval of unwanted memory items impairs their retention, as measured with intentional (directed) memory tests (p. 197).

*Intentional forgetting* (e.g., Johnson, 1994; Bjork et al., 1998) is defined as the motivated attempt to limit the future recall of a defined memory element. Not all memory items are welcome in awareness (Anderson and Hanslmayr, 2014), as we will address in the context of implementing routines in organizations that differ from previous ones.

Intentional forgetting serves a personal implicit or explicit motive (Bjork et al., 1998) or an individual or group-related goal (Harris et al., 2010). Many everyday situation require updating knowledge by exerting control over the memory (Aguirre et al., 2017). If a memory item is not helpful in a current situation, it should be intentionally forgotten, as it competes with the correct memory item that needs to be applied for goal directed behavior (Aguirre et al., 2017). Research has been shown that we are able to reduce the interference of unwanted memory items by making them less accessible (Aguirre et al., 2017).

Linked to this research are studies on *directed forgetting* as well as research addressing the forgetting of habits (Dreisbach and Bäuml, 2014) and social values (Isbell et al., 1998), which use explicit instructions to disregard given information (Golding and Long, 1998; Johnson, 1998; Kassin and Studebaker, 1998). The challenge inherent in disregarding given information is that it is also necessary to disregard all consequences for information processing, e.g., associations, as well as the coherence between cognitive elements (Johnson, 1998).

### The advantage of forgetting from the perspective of industrial and organizational psychology

From a task-related perspective, the main problem of a steadily growing amount of recallable knowledge is the decrease in the ability to effectively interpret information (O'Reilly, 1980; Huber, 1991). This is especially challenging for tasks which are non-routine (“not programmed decision,” Jones and Bouncken, 2008) and require complex problem solving and comprehensive decision-making processes that need to be solved under time pressure while considering several quality standards simultaneously (Eppler and Mengis, 2004; Paul and Nazareth, 2010; Netten and van Someren, 2011; Jackson and Farzaneh, 2012). As a result, the amount of recallable knowledge might suggest contradictory decision criteria or action steps, which might lead to cognitive dissonance and mental stress (Aikat and Remund, 2012). In such situations with a high amount of uncertainty and ambiguity, the amount of stored information does not lead to a reduction of uncertainty, but rather to an increase of uncertainty about which tasks need to be dealt with (Grote, 2009).

A high amount of recallable information will lead to difficulties in interpreting information and will impede the evaluation of alternative ways to reach strategic goals (Lipshitz and Strauss, 1997). This will also result in uncertainty about which goals can be achieved under consideration of all knowledge (Grote, 2009) or to problems in deriving connections and causalities (Kareev, 2000) and patterns among the noise of all of the available information.

The organizational psychology perspective additionally looks at technologies in organizations and the interplay between technology and people (Jones and Bouncken, 2008) in order to transform raw material into services and valuable goods and products (Emery, 1959). The recall of knowledge without limits, lead to the challenge that “more and more” recallable information will be a burden and a factor of strain, thus impeding efficiency and effectiveness (O'Reilly, 1980; Hwang and Lin, 1999; Edmunds and Morris, 2000; Eppler and Mengis, 2004; Bawden and Robinson, 2009; Bettis-Outland, 2012; Strother et al., 2012; Sabeeh and Ismail, 2013). An example of this is the productivity paradox (Dehning et al., 2003; Karr-Wisniewski and Lu, 2010) or the information paradox, which demonstrates that more information technology does not necessarily lead to increased productivity but can reduce productivity instead (Klausegger et al., 2007; Rajkumar et al., 2010; Ammu and Irfanuddin, 2013; Hunter et al., 2013).

### Knowledge management and forgetting

According to the organizations-as-brain metaphor (Morgan, 1998), organizations are information processing systems (Galbraith, 1977; Walsh and Ungson, 1991; Schilling and Kluge, 2004, 2013; Rebernik and Širec, 2007) which are capable of making information out of data, interpreting information (Daft and Weick, 1984), restructuring, storing and disseminating knowledge, and putting it into practice (Huber, 1991). Organizations process information and coordinate its application in order to achieve higher-order objectives (Huber, 2011; Nerdinger et al., 2011). In combination with the resource-based view on organizations (Wernerfelt, 1984), information is perceived as a resource (Wernerfelt, 1984, p. 172). In the last decades, the resource-based view has proposed the importance of acquiring, storing and disseminating information, and has investigated how:

- the underlying business processes should be designed most efficiently (Nonaka and Takeuchi, 1995; Capurro, 1998; O'Dell and Grayson, 1998; North, 2001; Reinmann-Rothmeier, 2001; Saint-Onge and Wallace, 2003; Heisig, 2005; Probst et al., 2005), and
- barriers to information acquisition and dissemination processes can be resolved (Schilling and Kluge, 2009).

The forgetting of information was not integrated in this research, and is regarded as a problem to be solved (Argote and Epple, 1990), as forgetting has been discovered to be a precondition for successful learning (Easterby-Smith and Lyles, 2011). Nevertheless, there is a gap in the research regarding appropriate methods and frameworks to support forgetting.

From a knowledge management perspective, the steadily growing amount of knowledge impedes the structuring and classification of that knowledge, meaning that it cannot be assigned to a specific context and loses its value for application (Martin de Holan and Phillips, 2004). Gronau (2009, p. 48) points to the necessity to renew knowledge, and to exchange and discard particular memory items from the organizational memory basis in order to cultivate organizational memory. Known ways to achieve this are to terminate communities (on a structural and process level) or to file and archive documents (on a technical level). No possibilities exist to utilize the potential of intentional forgetting for organizational knowledge management (Wenger et al., 2002; Bagherzadeh et al., 2010; Rezazade et al., 2011; Wolf et al., 2011; Argote, 2013; Eryilmaz, 2016).

In knowledge management, a possibility to link forgetting to business processes is to model business processes with a special focus on person-bound knowledge, which is fundamental to effectively and efficiently execute and perform a knowledge-intensive business process. Modeling approaches can be used for this purpose (Sultanow et al., 2012).

## What should be forgotten? routines as part of the organizational memory

This review builds on the assumption that organizations, as information processing systems, possess a memory that is comparable to the human memory (Hedberg, 1981; Daft and Weick, 1984; Huber, 1991; Walsh and Ungson, 1991). Organizational memory can be found in transformation processes (Walsh and Ungson, 1991), within which some form of input (e.g., material, energy, people, client orders) is transformed into output (e.g., products, services, employee skills, garbage). These transformation processes are mapped onto *routines* (Cyert and March, 1963). All routines taken together build a higher-order routine, which represents the capacity to build such transformation processes (Winter, 2003). Researchers investigating organizational routines (e.g., Gersick and Hackman, 1990; Becker, 2004; Miller et al., 2012; Helfat and Karim, 2014; Pentland and Hærem, 2015) or organizational forgetting (Martin de Holan and Phillips, 2004; Martin de Holan et al., 2004) stress the impact of routines on organizations' stability and lack of change. Nevertheless, it is not stated explicitly how this stabilization works or how the adaptation of routines through forgetting can be used to support change and adaptation to the environment. For instance, Tsang and Zahra (2008) and Miller et al. (2012) conclude that adaptation requires forgetting of routines (Miller et al., 2012, p. 1552) but do not elaborate on how an organization should accomplish this and do not provide a model which could be applied.

Organizational routines are “multi-actor, interlocking, reciprocally-triggered sequences of actions” (Cohen and Bacdayan, 1994, p. 554). As routines are the relevant source of stability, reliability and speed of organizational transformation processes, routines are central to our propositions, as they additionally serve purposes of information and knowledge storage (Cohen and Bacdayan, 1994; Becker, 2004). Routines enable coordination and controlling of actions and efficient use of cognitive resources, and reduce uncertainty while also constituting an essential part of organizational memory (Willke, 1998, p. 6). Cohen and Bacdayan (1994) see parallels between routines on an organizational level and procedural knowledge on an individual level as well as the distributive knowledge on a group level. Routines are comparable to distributed procedural memories (Cohen and Bacdayan, 1994), which are implemented into information systems in modern industries and organizations (D'Adderio, 2003).

The defining characteristics of routines are that they are repetitive, and consist of perceivable action patterns (Becker, 2004) and mutually dependent/interdependent actions, which are performed by several actors (Becker, 2004; Pentland and Hærem, 2015).

Organizational routines differ with respect to their content, structure, sequence in time, amount of formalization and the required knowledge (in terms of memory items) that need to be applied.

In the following, we address knowledge intensive routines, also called knowledge-intensive business processes (Gronau and Weber, 2004; Gronau et al., 2004; Lass et al., 2011; Gronau, 2012). A process is seen as knowledge-intensive if its value can only be created through the fulfillment of the knowledge requirements of the process participants. Clues that a process is knowledge-intensive include a large diversity of information sources and media types, a large number of process participants with different expert reports, the use of creativity, or a high degree of innovation and an available degree of scope for decisions (Gronau and Weber, 2004).

**Figure 2:**
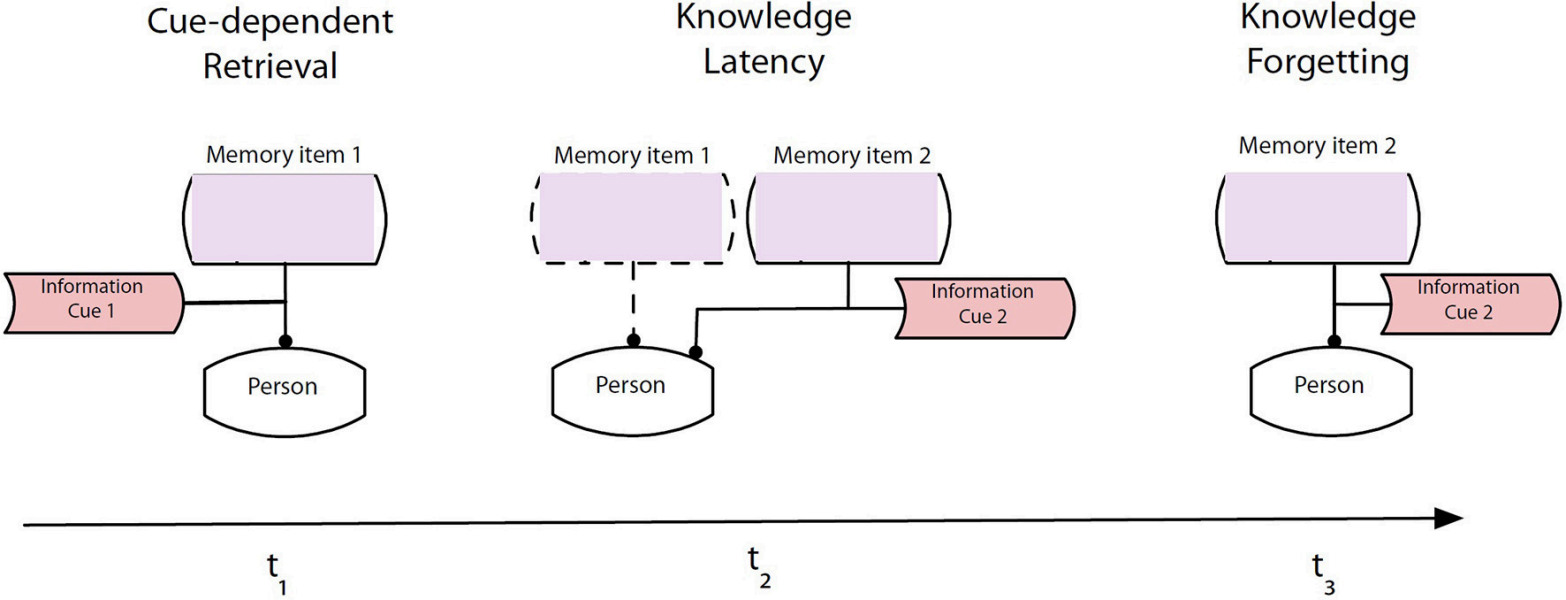
Phases of organizational forgetting, depicted in a KMDL activity model (see above).

Knowledge-intensive business processes are often depicted using the Knowledge Modeling and Description Language KMDL (Beckmann and Krause, 2013; Schmid and Kern, 2014; Neumann, 2015), which is used below to illustrate the idea of intentional forgetting (Figure 2). KMDL includes three “views” and an additional analysis and report functionality (Modelangelo, 2017). The process view of KMDL shows the business process with its tasks, roles and information systems, while the activity view shows where knowledge or information is converted by workers who perform a task. The activity view shows persons, activities related to business process tasks, person-bound knowledge and information, and visualizes the influence of forgetting on business processes with the appropriate level of detail.

The knowledge intensity of routines can be made visible through modeling techniques in process-related knowledge management (e.g., overviews by Gronau, 2012; Maasdorp and Gronau, 2016).

## The role of retrieval cues in organizational forgetting of routines

As outlined above, if environmental conditions change, humans adapt to these changes (Nairne and Pandeirada, 2008) by selecting new goals. Previously relevant goals need to be forgotten in order for persons to concentrate on new goals (Altmann and Gray, 2002; Roediger et al., 2010). In this respect, forgetting suppresses information that has become obsolete (Schooler and Hertwig, 2005).

In our propositions, retrieval theories are used to actively support forgetting. Retrieval theories explain forgetting in terms of cue overload, cue availability, consolidation and repression (Nairne and Pandeirada, 2008; Roediger et al., 2010; Gronlund and Kimball, 2013) and propose that recall is triggered by cues.

In terms of retrieval theories, forgetting is initiated by subsequent activity that might lead to weakening of the cue-target association (Nairne and Pandeirada, 2008, p. 186) or by cue overload (Nairne and Pandeirada, 2008; Roediger et al., 2010). The greater the number of memory items that are associated with a particular cue, the more overloaded it is and the less diagnostic value it has for recall. Moreover, if cues that are needed for recall are not present in a current situation, recall does not take place, and cue-dependent forgetting (Tulving, 1974) occurs. Forgetting results from a lack of retrieval cues (Nairne and Pandeirada, 2008). In organizations, retrieval cues include signs, order forms, rooms, persons, explicit instructions, user interfaces, work flow systems, or technical signals from machines.

If a particular cue is missing over a longer period of time, resulting in no recall of that cue, forgetting will begin, as the retrieval strength of the memory item associated with the retrieval cue is reduced (Bjork and Bjork, 1992, 2006; Bjork, 2009). The retrieval strength represents the accessibility of particular memory items. Accessibility can be differentiated in terms of storage strength and retrieval strength. The former describes the thoroughness with which a memory item is stored and anchored in memory. Memory items with high storage strength might have low retrieval strength due to longer periods of non-use. The new theory of disuse (Bjork and Bjork, 1992, 2006; Bjork, 2009, 2011) explains forgetting through a generally unlimited storage capacity of long-term memory and a limited recall capacity. At a certain point in time, only a limited number of items can be recalled. Whether or not a memory item is recalled depends on its retrieval strength. *As recall is cue-dependent, the absence of retrieval cues results in reduced retrieval strength*. If it is necessary to recall a particular memory item, this memory item must be discriminated from other items, which are likewise associated with that cue. How well and precisely items can be distinguished from each other depends on their retrieval strength (Bjork and Bjork, 1992). The retrieval strength of an item is relative to the retrieval strength of other items in memory, which are also linked to a particular retrieval cue. Upon recalling memory, items that were newly acquired in times of missing retrieval cues or information, their retrieval strength is increased, and the retrieval strength of the memory items that are not recalled is decreased (Bjork and Bjork, 1992, p. 43).

Based on the theories of retrieval strength outlined above, the elimination of these cues will enable the weakening of memory items and therefore forgetting insofar as the memory item is not activated because the related situational, sensory or routine-related cues are not present.

The relationship between forgetting and the intentional elimination of retrieval cues, and its impact on the actors in a routine, is as follows: Retrieval cues that are not (any longer) available play a central role in forgetting. If retrieval cues activate memory items with the highest retrieval strength, a strong variation of cues (also including the actors involved) leads to the fact that memory items to perform routines are no longer activated (Cohen and Bacdayan, 1994), are suppressed, and new and desired actions are performed instead.

In proposing forgetting as an activity in which retrieval cues are eliminated over time, forgetting can be conceptualized into three phases (Figure 2).

In state t_1_ the memory item 1 is recalled by using cue 1. Intentionally, in t_2_, the cue for memory item 1 is removed. Knowledge 1 is still available, but is fading due to reduced retrieval strength (see above). The recall of memory item 2 is triggered by presenting cue 2 in order to support the association between cue 2 and memory item 2. The authors call this the state of “knowledge latency.” This means that even under the condition of presenting cue 2, it is still possible to recall memory item 1, but it is less likely than recalling memory item 2, which possesses a learned association between memory item 2 and cue 2. In state t_3_, there is no recall of memory item 1. Instead, there is a solid recall of memory item 2. Therefore, it has been possible to forget memory item 1 in favor of memory item 2.

In this respect, an organization forgets because the actors in knowledge-intensive business process-related activities forget (Hedberg, 1981; Sinkula, 2002; Cegarra-Navarro and Moya, 2005; Becker et al., 2006; Zhao et al., 2013; Akhshik, 2014) and it becomes important to disconnect or suppress learned cues and action associations at a desired point in time (Hedberg, 1981).

## A cue classification for the elimination of cues

Transferring the findings on the effects of the elimination of retrieval cues which explain forgetting to an implemented process of intentional organizational forgetting, we propose that three cue types need to be considered as important in the forgetting of organizational routines and are directly related to the routine (Table 1):

- Sensory cues, which are the basal cues such as smell, taste, light, color, sound, tactile perceptions, temperature, or physical pain that trigger the recall of certain memory items (visual, olfactory, oral, tactile),
- Routine-related cues, which include actor-related, object-related, sequence of task-related and information-related cues, and
- Time and space cues, which include stimuli indicating location (e.g., production site) and time (of year, week, day) of the execution of the routine.

**Table 1:** Cue types, definitions and examples for organizational routines.

**Cue types and definition**	**Examples**
Sensory cues, -> basic physical stimuli and bodily perceptions	Smell, taste, light, color, sound, skin sensations (tactile perception), temperature, or physical pain
Routine-related cues, –>which are business process-related stimuli, and which in their different combinations form different routines in order to achieve different objectives	Team- and actor-related cues (manner of task coordination and orchestration), object-related cues (e.g., material and technical tools), sequence of task cues (e.g., orders from customers) Information cues (e.g., quality standards, production requirements, time limits, production goals),
Time and space cues, ->which are stimuli specifying space and time in which a routine is performed and which exist independently of the routine and add additional information to the execution of the routine	Location (e.g., of production and culture, e.g., Asia, the USA or Europe), time of year (season), time of week, time of day
Situational strength cues, ->defined as implicit or explicit cues representing the psychological pressure provided by external entities (e.g., supervisors) regarding the desirability of potential behaviors.	Clarity of psychological pressure, e.g., are the most salient cues eliminated or the weak ones? Consistency, e.g., are all cues eliminated that support forgetting of a particular routine or only some? Are old and new cues presented simultaneously? Consequences, e.g., are actors reinforced to recall a new routine and punished for recalling a to-be-forgotten routine? Constraints, e.g., are actors actively prevented from executing a routine that is defined as the to-be-forgotten routine, e.g., by technical means? This would mean that even if the to-be-forgotten routine is recalled and activated by some cue, its execution is technically not possible

Finally, we propose a fourth cue, which is a “meta-cue.” This describes the situation in an organizational change phase in which cues triggering the recall of a to-be-forgotten routine, and cues that are supposed to trigger the new routine that replaces the old routine, are simultaneously available. This situation could arise at t_2_ in Figure 2 (phase of knowledge latency) In the case of simultaneously available cues that activate both routines at the same time, actors in the organization need to decide which routine to execute and behave according to the psychological pressure caused by the situational strength (Meyer et al., 2010). Therefore, these cues are called:

- Situational Strength Cues, which include implicit or explicit cues provided by external entities (e.g., supervisors) regarding the desirability of potential behaviors (Meyer et al., 2010).

Situational strength (Meyer et al., 2010), which we assume to be less commonly known, is defined as “implicit or explicit cues provided by external entities regarding the desirability of potential behaviors” (p. 122). Situational strength results in a psychological pressure on the individual to show or not show particular behaviors. As early as the 1960s, Forehand and Vonhallergilmer (1964) described three options to affect employees' behavior: (1) to define stimuli, (2) to limit the freedom of behavior, and (3) by means of reinforcement or punishment.

Such an organization-related and content-related typology of cue types has not yet been defined, either in cognitive psychology or in organizational forgetting literature. So far, cues have been distinguished concerning, e.g., their specificity (Ellis, 1996), whether they represent a certain physiological state (state-dependent retrieval, Aggleton and Waskett, 1999), whether they are common or rare, abstract or concrete, whether they are rich or poor contextual cues, object-related or emotion-related, pictorial or verbal (Tulving and Thomson, 1973; Keller, 1987; Ellis, 1996; Aggleton and Waskett, 1999; Dumais et al., 2016; Uzer, 2016). These categories emerge from experimental investigations from either basic laboratory or advertising research.

**Figure 3:**
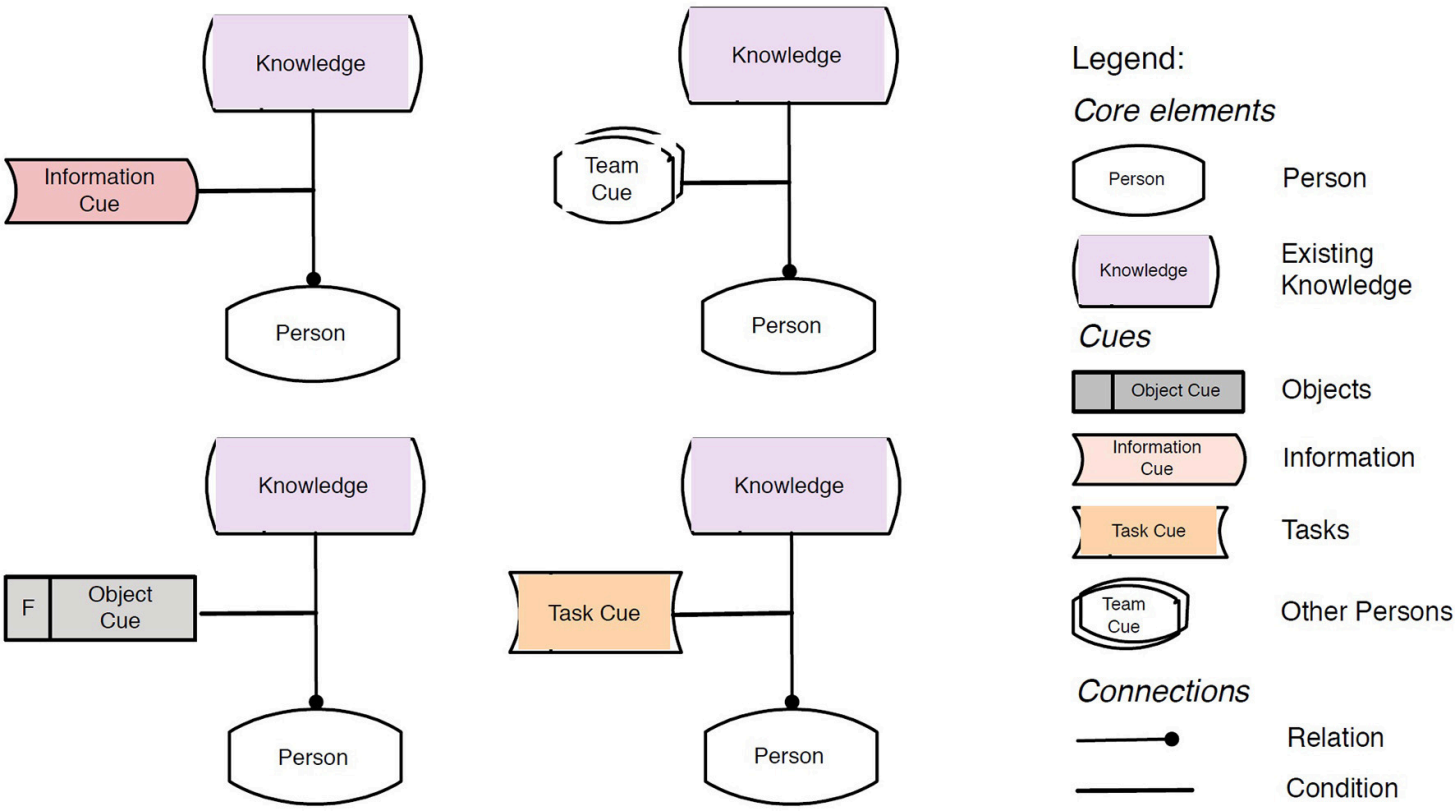
Routine-related cues. Syntax: KMDL (Maasdorp and Gronau, 2016, p. 29).

For the organizational context of forgetting, we propose a distinction between sensory, routine-related and situation cues in order to best match the definition of routines defined as “multi-actor, interlocking, reciprocally triggered sequences of actions” (Cohen and Bacdayan, 1994), which encompass process-related memory items that have been acquired during an initial learning and training phase and by training and learning on the job. The context of the specific phase provides cues, which have to be present in order to recall the memory items. These cues can consist of external objects related directly to the task (e.g., customer orders, technical drawings, production requirements etc.) or to the process environment in general, such as other contributing actions and team members. These cues trigger sequences of actions and the recall of the necessary memory items. Figure 3 describes the routine-related cues using the modeling language KMDL.

## Propositions on the elimination of cues to support forgetting

Retrieval theories led us to conclude that the elimination of retrieval cues supports the forgetting of routines on an individual and group level.

*Generally, it can be assumed that sensory cues and routine-related retrieval cues, i.e., information cues, object cues, task cues and actor cues, space cues and time cues, of an old routine, need to be eliminated in order to stop the recall and retrieval of the old routine*.

**Figure 4:**
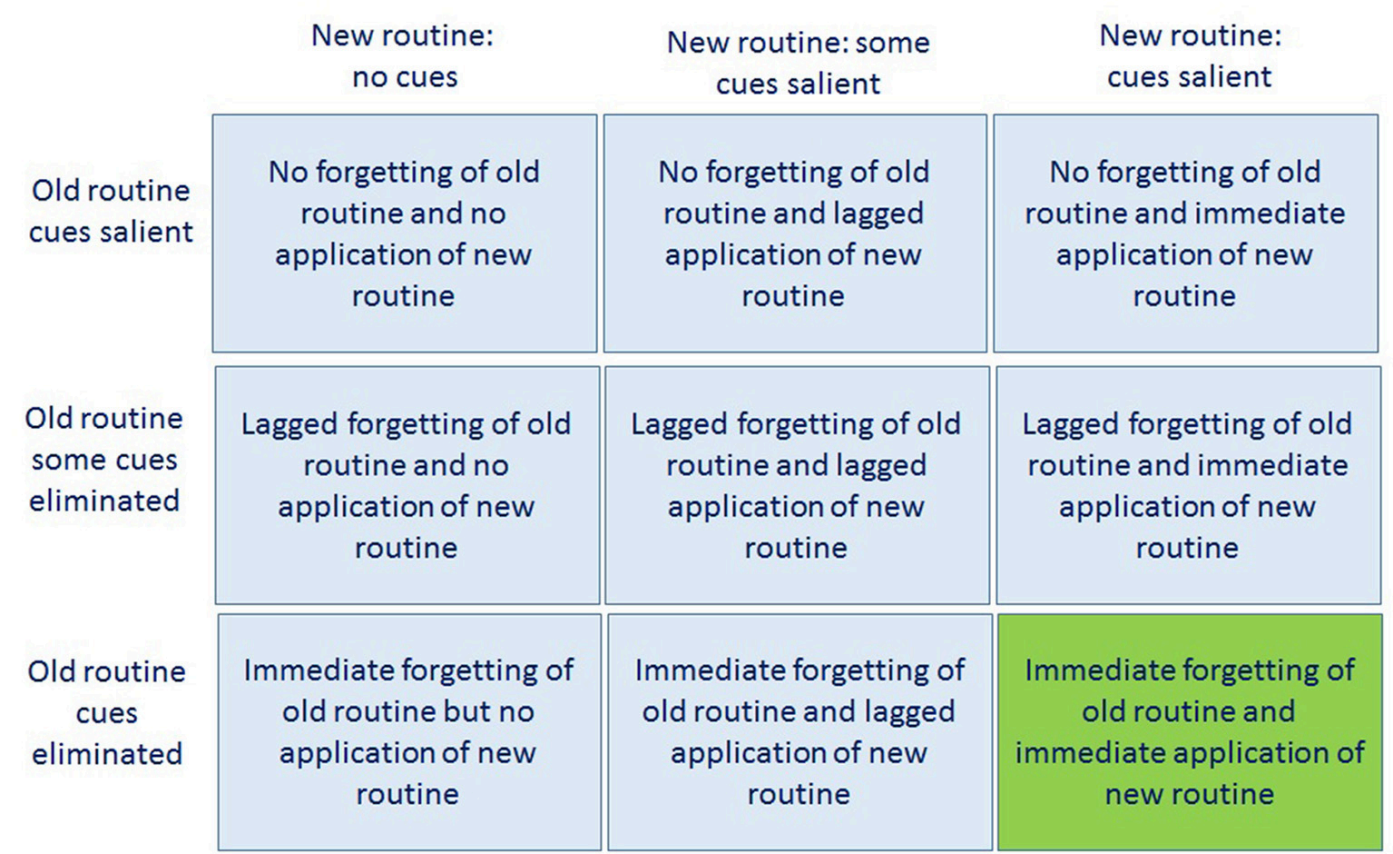
Combinations of cue elimination and presence of old routine and of new routine.

An extreme example of the elimination of all cues would be the closing down of a plant in which a product was manufactured according to an old routine A (elimination of all cues) and the manufacturing of the same updated product line according to a new routine A+ in a newly built production site in a different location with a rearranged actor composition. In an abstract manner, Figure 4 shows the different combinations possible for the availability of cues of the old and new routine. The ideal situation for fast forgetting would be the one depicted in the bottom-right box in Figure 4 (fast forgetting of old routine and immediate application of new routine), as argued above.

As this ideal model of radical elimination of all cues associated with the old routine seems rather unlikely in all change processes from old to new routines, research is needed to address the question of which cues have the strongest impact on forgetting in order to select those cues for elimination. So far, cues have been investigated as triggers of unwanted activation of routines that should be forgotten, or as triggers for the subsequent action in a multi-actor routine (Pentland and Hærem, 2015). In the present paper, we focus on two cue types: first, the actor and group composition cues, as from an ethical and human resource perspective, workers and employees should not be “eliminated” and second, the situational strength cues as outlined below.

We built the following propositions based on the approach by Meyer et al. (2010) and therefore start with “situational strength” cues (clarity, consistency, consequences, constraints) which are relevant for phase 2 (see Figure 2, at t_2_ phase of knowledge latency).

*Clarity* means the degree to which salient cues related to routines and their affordances are present, e.g., through information cues, object cues, action sequence cues and team members, as well as support by supervisors. As described above, organizational forgetting means the elimination of cues that are linked to the routine that is supposed to be forgotten. At the same time, cues that support the new routines need to be made salient (see Figure 4). Situations become strong if all cues leading to recall of the to-be-forgotten routine are eliminated and the cues leading to recall of the new routine are made maximally salient. Situations are weak if cues of the old routine are still present and cues of the new routine are not, or if cues that recall the old and the new routine are present simultaneously.

*Proposition 1: The forgetting of routines is supported by eliminating all salient retrieval cues that can activate the to-be-forgotten routine and by making cues that enhance the execution of the new routine maximally salient*.

*Consistency* means the degree to which cues are compatible with work-related affordances, e.g., a high production output. It is often likely that organizations will implement a sequence of action-related cues that activate a new routine, but that simultaneously, other cues, e.g., concerning timely production, quality or efficiency (included in the information cues) can no longer be met. Such an inconsistent cue configuration leads to a “mixed message” about how to behave and which routine to execute. Inconsistency and mixed messages lead to time pressure, because the new activity cannot be executed as smoothly and quickly as the activity that needs to be forgotten. A study by Betsch et al. (1999) showed that time pressure supports the relapse to old routines and facilitates non-forgetting (Betsch et al., 1999; Becker, 2004). Under time pressure, there is a decrease in the speed of learning (“power law of practice”; Proctor and Dutta, 1995; VanLehn, 1996; Bourne and Healy, 2012) to execute a new routine and in the speed of forgetting of what is supposed to be forgotten: The inconsistent cue configuration reinforces the application of the old routine that is faster to perform. At the same time, the parallel demand to execute the new activity leads to punishment, as goals cannot be met. This facilitates the relapse into the activity that needs to be forgotten.

*Proposition 2: A main reason for the perception of inconsistency in organizations is time pressure. Forgetting of routines is supported by eliminating time pressure while performing the new routine*.

*Consequences* describe the extent to which decisions and actions lead to positive or negative consequences. The failure to forget an activity should be punished (from a learning psychology perspective) and the application of the new activity should be reinforced (Newstrom, 1983). This principle can be applied on an individual and group level. However, the opposite is often the case, namely that the to-be-forgotten activity is reinforced (Becker, 2004), and the routine is therefore retained and continuously performed. At the same time, the execution of the new routine is punished, for instance because individuals feel less competent as the technical systems shows errors; they feel a loss of control, frustration and a perceived decrease in performance (at least in the short term, Lazaric and Denis, 2005).

*Proposition 3. Forgetting of routines is supported by punishing the execution of the to-be-forgotten activity while simultaneously reinforcing the execution of the new activity*.

*Constraints* describe the extent to which individual freedom of decisions or actions are controlled externally. For example, in many cases, an organization allows individuals and groups to use the to-be-forgotten routine and the new routine in parallel, simply because it is technically possible, for instance because the old (to-be-forgotten) software has not been deleted from a computer. In such cases, retrieval cues are not eliminated and forgetting is impeded (Besnard and Cacitti, 2005). With respect to computer- and IT-based workplaces, Besnard and Cacitti (2005) showed that the forgetting of an old routine is hindered if the interface does not eliminate the cues that belong to the to-be-forgotten activity, and therefore does not suppress its execution.

*Proposition 4. Forgetting of routines is supported by actively constraining the execution of the to-be-forgotten activity*.

So far, we have addressed the consequences of combining cues from old and new routines, which can be summarized using the term situational strength cues. We now address actor-related cues. As organizational forgetting is assumed to take place through a combination of forgetting on the individual, group and organizational level (Sinkula, 2002; Cegarra-Navarro and Moya, 2005; Becker et al., 2006; Zhao et al., 2013; Akhshik, 2014), one should also consider the link between individual- and group-level forgetting. Three general assumptions concerning the relationship between individual- and group-level forgetting can be found in the literature:

*Proposition 5: Individual forgetting of business process-related activities is a precondition for group-level forgetting*.

This proposition is based on the work of Akhshik (2014), Becker et al. (2006), Cegarra-Navarro and Moya (2005), Sinkula (2002), and Zhao et al. (2013). Routines are patterns of actions of several actors (Pentland and Hærem, 2015) and a network of functional events which are given a direction based on the sequence and defined order of these actions (Pentland and Hærem, 2015). Therefore, it stands to reason that individual forgetting is a precondition for the forgetting of a business process that is executed by a group. Additionally, it is assumed that the speed of group-level forgetting depends on the speed of individual forgetting.

*Proposition 6: Forgetting of business process-related activities on the group level takes longer than forgetting on the individual level (Akgün et al., 2006)*.

This proposition is grounded in the argument that the action of one actor triggers and initiates the subsequent action of the next actor with a cue. With respect to retrieval cues, it is assumed that group-level forgetting takes place more slowly, as the interaction between group members sets retrieval cues. The actors in the group are retrieval cues themselves, as they initiate a subsequent action by the next actor. The forgetting of routine actions that are executed by a large number of actors and which need to be forgotten by all actors will therefore take longer than the forgetting of routine actions that are only executed by a small number of actors.

*Proposition 7: Forgetting of business process-related activities can be accelerated if group composition is altered and memory elements are eliminated completely (Klein, 1989)*.

According to Klein (1989), forgetting can be achieved by means of replacement on the management level, e.g., if top managers and supervisors are replaced in order to forget practices which have proven to be unsuccessful and undesirable. This proposition is also supported by Gorman and Cooke (2011) and Cooke et al. (2013), who investigated interactive team cognition. Their studies showed that skill decay on a group level is predicted less by the rate of individual forgetting, and more by considering measures of group interaction and a poorer coordination as a result of forgetting. In summary, it is assumed that on a group level, it is necessary to forget not only the knowledge about the business process but also the memory items concerning the coordination of the activities.

## Conclusions

The purpose of our article was to promote the concept of forgetting as a beneficial human memory process to adapt to new situations for use in organizations, and to transfer the principles of cue-dependent retrieval to support faster organizational forgetting. Our approach to forgetting in organizations proposes processes that deliberately impede the recall of knowledge and organizational memory items. Accordingly, in the case of a particular query, these memory items and information elements are not provided, thus supporting an organization's changed strategic goal achievement.

Our review concerning the concept of forgetting using cue elimination opens up a new paradigm for organizational change and learning. The use of retrieval theories and the elimination of retrieval cues in order to actively manage forgetting makes the hitherto abstract concept and ideas of organizational forgetting, and the necessity thereof, operational and applicable. Nevertheless, to empirically test our assumptions, extensive experimental research will be necessary to find out more about the opportunities and boundaries of this paradigm. For instance, we propose that the use of cues for old and new routines simultaneously will lead to weak situational strength due to low clarity and low consistency of cues available in the situation.

### Implications for further research

Although the term forgetting has been used in organizational science for some time now, the underlying forgetting processes have not been described in detail. We presented the state of the art of forgetting research in order to infer mechanisms that can also be used for purposes of implementation in organizations, when forgetting is required for adaptation to new environmental conditions. We would like to encourage researchers from organization science, organization development and Change Management to investigate the relevance of different cues (e.g., sensory, routine-related, and time-and-space cues) but also the relevance of meta-cues in such transition processes.

For instance, basic research could experimentally test sensory cues in a lab-based setting, while routine-related cues (actor, sequence of action, object, information cues) need a more complex simulation of production contexts to investigate the effect of the presence and absence of old and new cues. These complex production simulations can be found in so-called “learning factories,” which are mostly located in universities (in Germany) and were originally developed to teach students about process improvement (Prinz et al., 2016). The advancements of learning factories over the past years show that they can be used to impart knowledge about very different topics. Abele and Metternich defined five topics to be learned in a learning factory: production processes, logistic processes, energy efficiency, design processes, virtual/ digital/ organizational change (Abele and Metternich, 2015). Currently, managers, shop floor workers, and workers in planning and control are also important target groups (Kreimeier et al., 2014; Prinz et al., 2016; Gronau et al., 2017). The option to engage in production processes within a real-world manufacturing environment allows one to transfer problems, e.g., of forgetting and adapting, to one's own operational challenges. This can also be used for research purposes and for evidence-based decision making regarding which cues need to be considered more strongly and which are more or less irrelevant. In this setting, one can apply experimental designs in which the speed of forgetting can be investigated by manipulating cues and cue configurations (Vladova et al., 2017).

Further research could additionally aim to discern methods to model the forgetting process. A time-based model of forgetting that takes into account the effect of the different cue types is helpful in order to describe where, when and why forgetting takes place and how long this process takes, e.g., depending on the type of knowledge or routine.

### Implications for organizational practice

We argued that organizations are faced with the challenge of thinking about strategies on how to manage the increasing amount of data that are recallable in organizational information and memory systems. We suggested that organizations should adapt to the way in which human memory works, as human memory is likewise able to store a large amount of information. The advantage of the human memory system lies in its flexibility to react to a changing environment in which different cues and signals are present. If cues are absent or are eliminated, human memory “learns to forget” in terms of decreasing the retrieval strength of memory items associated with these cues.

Organizations that are confronted with change and development processes can make use of these mechanisms in their change management programs and interventions. Facing change does not solely mean creating a vision, communicating the vision, training and enabling workers to behave according to the vision, and reinforcing and institutionalizing new routines, as is the case in many change management concepts (e.g., based on Kotter, 1998). Rather, it also means removing all cues that might recall old routines and habit patterns. Only addressing the new routines while cues recalling old routines are still present will lead to a lack of clarity, inconsistency and mixed messages. This in turn will lead to a low situational strength and uncertainty about what the organization actually expects workers to do. In this regard, we propose that change management agents also need to put effort (and resources) into eliminating retrieval cues of old routines.

With respect to actor and team cues, the research we analyzed suggests that when the aim is to support forgetting, one should also consider changes in actor and team composition. As actors are cues in a multi-actor routine, the exchange of an actor in a team and the “elimination” of a former team member (in this case meaning a relocation of an actor who will then join a different group of actors) would also support forgetting. In routines, which are multi-actor, interlocking, reciprocally triggered sequences of actions, actors serve as cues too, and should be considered in their role of a “cue” to recall memory items, which should be forgotten. Here, Human Resource Management and personnel planning systems and relocation become an issue.

We believe that the concept of forgetting holds more potential for empirical research than has thus far been exploited. Forgetting and unlearning has a long tradition in terms of theoretical discussions of its value for organizational learning and change, but so far, it has shown no concrete impact, as empirical findings are lacking. With our review, we identified a viable way to operationalize forgetting processes that can be manipulated by researchers in order to support practitioners in their change endeavors.

## Author contributions

AK wrote the article as the lead author. The literature review has been conducted by her. The article idea and the concepts are developed by AK and NG. The review is based on a successful proposal to the Deutsche Forschungsgemeinschaft and has been up dated in the phase of the article preparation. NG wrote the sections on knowledgement and business processes and explains the KMDL Modeling language. Propositions were developed to gether. The propositions are part of an innovative theoretical approach in combining organizational learning, unlearning, and intentional forgetting with basic research from cognitive psychology on intentional forgetting.

### Conflict of interest statement

The authors declare that the research was conducted in the absence of any commercial or financial relationships that could be construed as a potential conflict of interest.
